# Multicenter study comparing outcomes of robotic versus video-assisted thoracoscopic resection of non-small cell lung cancer following neoadjuvant chemoimmunotherapy

**DOI:** 10.1007/s11701-026-03326-4

**Published:** 2026-04-02

**Authors:** Shachi Srivatsa, Hadrien Maréchal, Nasser K. Altorki, Nestor Villamizar, Joseph D. Phillips, Philipp Schnorr, Dan Jones, Desmond D’Souza, Ioana Baiu, Mahmoud Abdel-Rasoul, Joachim Schmidt, Dao Nguyen, Robert E. Merritt, Jean-Marc Baste, Peter J. Kneuertz

**Affiliations:** 1 Division of Thoracic Surgery, Department of Surgery, The Ohio State University Wexner Medical Center, N846 Doan Hall, 410 W. 10th Ave, Columbus, OH 43210 USA; 2 Department of Thoracic and Cardiac Surgery, Rouen University Hospital, Rouen, France; 3 Department of Cardiothoracic Surgery, Weill Cornell Medicine/New York-Presbyterian Hospital, New York, NY USA; 4 Division of Cardiothoracic Surgery, The DeWitt Daughtry Family, Department of Surgery, University of Miami Miller School of Medicine, Miami, FL USA; 5 Section of Thoracic Surgery, Department of Surgery, Dartmouth Health, Geisel School of Medicine at Dartmouth, Lebanon, NH USA; 6 Division of Thoracic Surgery, Department of General, Visceral, Thoracic and Vascular Surgery, University Hospital Bonn, Bonn, Germany; 7 Department of Thoracic Surgery, Helios Hospital Bonn/Rhein-Sieg, Bonn, Germany; 8 Center for Biomedical Informatics, The Ohio State Wexner Medical Center, Columbus, OH USA

**Keywords:** Neoadjuvant, Immunotherapy, Checkpoint inhibitor, Robotic, Video-assisted thoracoscopic, Outcomes

## Abstract

**Objective:**

We sought to compare outcomes of robotic-assisted (RATS) and video-assisted thoracoscopic (VATS) resection of non-small cell lung cancer (NSCLC) after neoadjuvant chemoimmunotherapy.

**Methods:**

A retrospective database of patients treated with neoadjuvant chemoimmunotherapy followed by resection for NSCLC after between 2019 and 2025 at six centers in the US, France and Germany was analyzed. Patients undergoing RATS and VATS were included. Patient selection, extent of resection, pathologic and short-term outcomes were compared by operative approach. Inverse probability of treatment weights (IPTW) based on propensity scores were applied to balance the groups for outcome comparisons.

**Results:**

A total of 276 patients were included, of which 243 (88.0%) were approached by RATS and 33 (12.0%) VATS. Baseline characteristics were similar in both cohorts. Bronchoplasty (*n* = 8) or angioplasty (*n* = 6) were performed only using RATS. Conversion to thoracotomy was significantly higher with VATS (27.3% vs. 5.8%, *p* < 0.001). Oncologic outcomes, including R0 resection, lymph node harvest, major pathologic response, and pathologic complete response, were comparable. Overall complications were similar (33.3% vs. 35.8%, *p* = 0.78), but VATS had higher rates of major complications (21.2% vs. 7.8%, *p* = 0.01), respiratory failure (12.1% vs. 3.7%, *p* = 0.03), and unplanned reoperation (12.1% vs. 0.8%, *p* < 0.001). IPTW-adjusted analyses confirmed lower conversion (26.1% vs. 7.2%, *p* = 0.005) and major complication rates (22.7% vs. 7.4%, *p* = 0.01) with RATS, with no differences in overall complications (34.5% vs. 35.8%, *p* = 0.91) or length of stay (5.0 vs. 4.6 days, *p* = 0.46).

**Conclusions:**

Both RATS and VATS are feasible approaches for NSCLC resection following neoadjuvant chemoimmunotherapy and achieve comparable oncologic outcomes. However, RATS was associated with substantially lower conversion to thoracotomy and fewer major postoperative complications, while maintaining equivalent resection quality. These findings support an expanded role for robotic-assisted resection in the management of appropriately selected patients after neoadjuvant chemoimmunotherapy.

**Graphical abstract:**

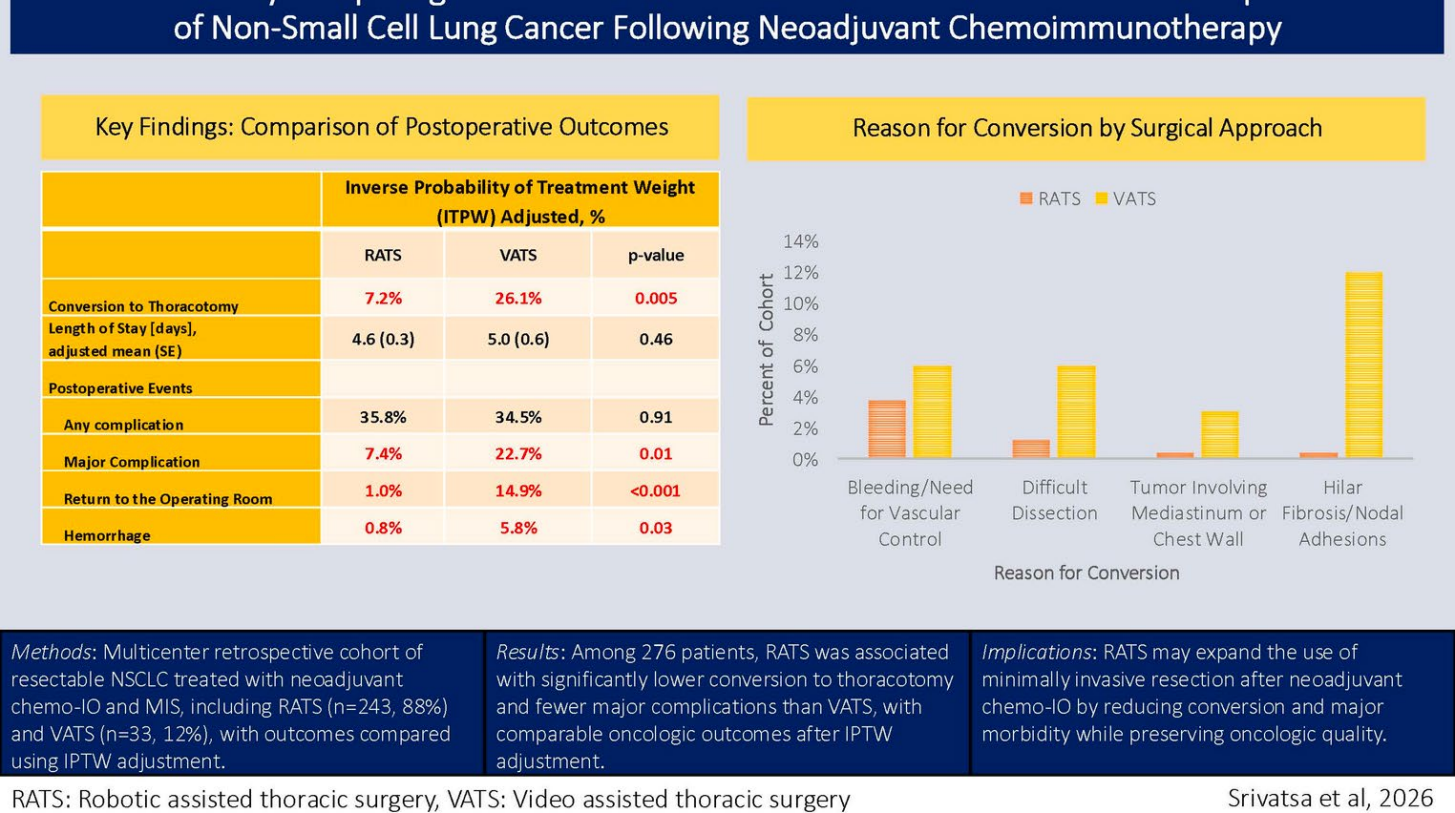

**Supplementary Information:**

The online version contains supplementary material available at 10.1007/s11701-026-03326-4.

## Introduction

Neoadjuvant or perioperative chemoimmunotherapy with immune checkpoint inhibitors (ICI) has been established as the recommended treatment for patients with operable locoregionally advanced non-small cell lung cancer (NSCLC) without actionable driver mutations [1, 2]. Multiple randomized phase III trials have demonstrated high rates of pathologic response, and long-term results of the CheckMate 816 trial have confirmed durable disease control and an overall survival advantage over chemotherapy with an impressive 5-year survival of over 90% amongst patients with a pathologic complete response [3–5].

At the same time, neoadjuvant chemoimmunotherapy has also created new technical challenges for surgeons. Immunologic treatment changes such fibrosis in the hilum and mediastinal lymph nodes as well as adhesions due to pneumonitis can make resections more difficult and push the boundaries of minimally invasive surgery [6, 7]. In clinical trials, most patients were treated with a conservative open thoracotomy approach, with frequent conversions in patients selected for video-assisted thoracoscopic surgery (VATS) [4, 8]. Subsequent observational studies have demonstrated higher success rates with minimally invasive surgery after neoadjuvant chemoimmunotherapy, which frequently utilize robotic-assisted thoracoscopic surgery (RATS) [9–13].

We hypothesized that RATS would facilitate complex resections following neoadjuvant chemoimmunotherapy while maintaining comparable perioperative and oncologic outcomes relative to VATS.  The objective of this study was to compare outcomes between RATS and VATS following chemoimmunotherapy for NSCLC in centers with expertise in centers minimally invasive surgery.

## Methods

### Data source and patient selection

This is a retrospective review of a multicenter database of patients undergoing NSCLC resection after chemoimmunotherapy at six contributing academic medical centers, including The Ohio State University Wexner Medical Center (Columbus, OH, USA), Rouen University Hospital (Rouen, France), New York Presbyterian Weill-Cornell Medical Center (New York, NY, USA), University of Miami Miller School of Medicine (Miami, FL, USA), Dartmouth Heath (Lebanon, NH, USA), and University of Bonn (Bonn, Germany). The study was approved by the respective Institutional Review Board at each institution. Patient consent was waived due the retrospective design with minimal risk to participants (OSU STUDY20250691). Centers provided data as specified through a menu-driven database, prepared with precise coding instructions, dropdown lists, and data definitions.

Patients undergoing resection by either RATS or VATS after chemoimmunotherapy with ICI between years 2019–2025 who had complete data on surgical approach and outcomes were included. The approach was selected at the discretion of the surgeon. Three of the five centers performed both RATS and VATS, while two centers performed RATS only. The decision between VATS and RATS was primarily based on access to the robotic system or preference. RATS was performed with the da Vinci (Intuitive Surgical, Inc, Sunnyvale, CA, USA) surgical systems using four ports and an optional assistant port (RPL-4). The VATS approach was defined as any video-assisted technique without rib spreading and included uniportal and multiportal approaches.

Consecutive patients meeting inclusion criteria during the study period were included from each participating center. No patients were excluded on the basis of demographic characteristics, comorbidities, or tumor stage. Standardized data definitions and coding instructions were distributed to all centers to minimize inter-institutional variability in data capture. Patients who underwent planned thoracotomy (*n* = 44) were excluded. The extent of resection was based on the tumor involvement. A parenchyma sparing strategy was employed for patients with borderline lung function. We also excluded patients who were deemed unresectable based on imaging or surgical exploration (*n* = 7). One patient was excluded based on Pancoast tumor, and ten patients for lacking pathology data. We also excluded patients who received preoperative ICI alone (*n* = 8), chemotherapy alone (*n* = 2), or radiation (*n* = 3).

## Definitions

Staging information was provided according to the AJCC ninth edition staging manual. Clinical TNM stage was determined based on CT and hypermetabolic activity on positron emission tomography (PET) scan. Invasive preoperative nodal staging by endobronchial ultrasound (EBUS) or mediastinoscopy was performed selectively. ICI agent, numbers of cycles administered, and time from last ICI dose to surgery in days was recorded. Restaging was routinely performed for all patients using CT scan, and PET-CT scan for selected patients. Pathology reports were reviewed to extract margin status and histologic tumor features. Response in the primary tumor was recorded in percent of remaining viable tumor, necrosis or fibrosis. Major pathologic response (MPR) was defined as equal or less than 10% of viable tumor in the surgical specimen. Pathologic complete response (pCR) was defined as the absence of any viable tumor or lymph node disease. R0 resection was defined as the absence of viable tumor in any margin, including parenchymal, bronchial and vascular margins. Number of lymph nodes resected and lymph node status was assessed for all resected lymph node stations.

## Outcomes

Unplanned conversion from RATS or VATS to open thoracotomy and reason for conversion was the primary endpoint. Resection margin and lymph node harvest number of stations and nodes were secondary oncologic outcomes. Length of hospital stay, postoperative events and 60-day mortality were secondary endpoints. Outcomes were defined based on the Society of Thoracic Surgery General Thoracic Surgery Database Version 2.4 training manual. Major complication was defined as any occurrence of pneumonia, respiratory failure requiring reintubation, myocardial infarction, pulmonary embolus, hemorrhage requiring reoperation or any return to the operating room.

### Statistical analysis

Patient, disease characteristics and outcomes were compared between RATS and VATS patients. Categorical variables were summarized as frequencies and percentages and compared between surgery groups using χ^2^ tests or Fisher’s exact tests where relevant. Continuous characteristics were reported as medians with interquartile rage (IQR) or means with standard deviation (SD) and compared using the Mann-Whitney-U test or student t-test as appropriate. Postoperative outcomes were compared following inverse probability of treatment weighing (IPTW) to balance the baseline characteristics between groups, as previously described [14]. IPTWs were estimated using multinomial logistic regression analysis including differential characteristics (*p* ≤ 0.2), including age, body mass index (BMI), forced expiratory volume in one second (FEV1), performance status, coronary artery disease, diabetes, clinical T-stage, and extent of resection. Adjusted outcomes were compared between surgery groups after adjusting for IPTWs using Rao-Scott chi-square (χ^2^) tests. Hypothesis testing for main outcomes was conducted at a 5% type I error rate. The statistical analysis was designed and conducted by an experienced biostatistician (MA) using SAS version 9.4 (SAS Institute, Cary NC) and Stata version 13 (StataCorp, College Station, TX).

## Results

A total of 276 patients were included, of whom 243 (88.0%) underwent RATS and 33 (12.0%) underwent VATS. Baseline demographics were generally similar between groups, including age, sex, body mass index, smoking status, and comorbidities (Table 1). VATS patients were more likely to have ECOG performance status of 0 (84.8% vs. 54.4%, *p* = 0.004). The median PET SUVmax was higher in the VATS group compared to RATS (16.2 vs. 8.7, *p* = 0.001). Preoperative mediastinal staging with EBUS was more common in RATS patients (85.9% vs. 67.7%, *p* = 0.01). Clinical stage distribution and induction treatment were similar between groups. Most patients had no clinical metastases at diagnosis: 217 (98.2%) of RATS and all 28 (100%) of VATS cases were M0 (*p* = 0.47). All patients received neoadjuvant chemoimmunotherapy. Nivolumab was administered in 132 (58.9%) of RATS cases and 24 (72.7%) of VATS cases; pembrolizumab was used in 64 (28.6%) and 4 (12.1%) patients, respectively. Other agents were used in 28 (12.5%) robotic and 5 (15.2%) VATS cases (*p* = 0.14). The interval from neoadjuvant therapy to surgery was slightly longer in the RATS cohort (median 43 days vs. 33 days for VATS, *p* = 0.01), but all patients proceeded to resection within a few months of induction. The observed platform distribution reflects institutional adoption strategies during the study period. Two centers had implemented a predominantly robotic minimally invasive approach for post-induction cases, whereas others utilized both RATS and VATS based on platform availability and surgeon preference. Most patients underwent lobectomy (88.5% RATS vs. 78.8% VATS), while pneumonectomy was performed more frequently in the VATS group (12.1% vs. 3.3%) (Table 2).

**Table 1:** Patient-, disease and treatment characteristics comparing RATS and VATS approach

	All (*N* = 276)	RATS (*N* = 243)	VATS (*N* = 33)	*p*-value
**Patient characteristics**				
Age [years], median (IQR)	65.0 (60.0, 71.0)	65.0 (59.0, 71.0)	66.0 (61.0, 73.0)	0.34
Gender				
Female	118 (42.8%)	101 (41.6%)	17 (51.5%)	0.28
Male	158 (57.2%)	142 (58.4%)	16 (48.5%)	
Smoking status				
Never	23 (8.4%)	21 (8.7%)	2 (6.1%)	
Former	197 (71.6%)	173 (71.5%)	24 (72.7%)	0.87
Active	55 (20.0%)	48 (19.8%)	7 (21.2%)	
BMI [kg/m^2^], median (IQR)	27.1 (23.5, 30.9)	27.1 (23.5, 31.0)	27.8 (23.5, 30.5)	0.93
ECOG Performance Status				
0	158 (58.1%)	130 (54.4%)	28 (84.8%)	**0.004**
1	103 (37.9%)	98 (41.0%)	5 (15.2%)	
2–3	11 (4.0%)	11 (4.6%)	0 (0.0%)	
Rheumatoid Arthritis	5 (2.7%)	4 (2.5%)	1 (4.0%)	0.67
Diabetes Mellitus	51 (18.5%)	48 (19.8%)	3 (9.1%)	0.14
Coronary Artery Disease	50 (18.2%)	43 (17.8%)	7 (21.2%)	0.63
History of Tuberculosis	5 (2.7%)	5 (3.1%)	0 (0.0%)	0.37
FEV1 [% predicted], mean (SD)	85.5 (18.38)	85.5 (18.69)	85.3 (16.25)	0.99
DLCO [% predicted], mean (SD)	72.1 (18.04)	72.1 (18.17)	72.4 (17.43)	0.75
**Disease Characteristics**				
Tumor Location				
RUL/RML	98 (35.5%)	85 (35.0%)	13 (39.4%)	
RML	11 (4.0%)	10 (4.1%)	1 (3.0%)	
RLL/RML	58 (21.0%)	54 (22.2%)	4 (12.1%)	0.75
LUL	67 (24.3%)	57 (23.5%)	10 (30.3%)	
LLL	39 (14.1%)	34 (14.0%)	5 (15.2%)	
LUL/LLL	3 (1.1%)	3 (1.2%)	0 (0.0%)	
Centrality				
Peripheral	108 (52.9%)	89 (51.4%)	19 (61.3%)	0.31
Central	96 (47.1%)	84 (48.6%)	12 (38.7%)	
PET-scan				
Tumor SUVmax, median (IQR)	9.6 (4.2, 18.2)	8.7 (3.7, 15.9)	16.2 (9.4, 24.5)	**0.001**
Tumor PD-L1 Expression				
<1%	91 (37.1%)	79 (37.1%)	12 (37.5%)	
1–49%	64 (26.1%)	56 (26.3%)	8 (25.0%)	0.99
>=50%	90 (36.7%)	78 (36.6%)	12 (37.5%)	
Invasive Mediastinal Staging				
EBUS	167 (83.1%)	146 (85.9%)	21 (67.7%)	**0.01**
Mediastinoscopy	14 (6.9%)	13 (7.6%)	1 (3.2%)	0.38
Clinical T-stage				
cT1	73 (27.4%)	67 (28.8%)	6 (18.2%)	0.14
cT2	97 (36.5%)	86 (36.9%)	11 (33.3%)	
cT3	70 (26.3%)	56 (24.0%)	14 (42.4%)	
cT4	26 (9.8%)	24 (10.3%)	2 (6.1%)	
Clinical N-Stage				
cN0	101 (38.1%)	89 (38.4%)	12 (36.4%)	0.86
cN1	62 (23.4%)	53 (22.8%)	9 (27.3%)	
cN2a	76 (28.7%)	68 (29.3%)	8 (24.2%)	
cN2b	26 (9.8%)	22 (9.5%)	4 (12.1%)	
Clinical M-Stage				
cM0	245 (98.4%)	217 (98.2%)	28 (100.0%)	0.47
cM1	4 (1.6%)	4 (1.8%)	0 (0.0%)	
**Treatment Details**				
Checkpoint Inhibitor				
Nivolumab	156 (60.7%)	132 (58.9%)	24 (72.7%)	0.14
Pembrolizumab	68 (26.5%)	64 (28.6%)	4 (12.1%)	
Other	33 (12.8%)	28 (12.5%)	5 (15.2%)	
Unknown	19 (6.9%)	19 (7.8%)	0 (0.0%)	
Number of cycles, median (IQR)	3.0 (3.0, 4.0)	3.0 (3.0, 4.0)	3.0 (3.0, 4.0)	0.39
Days from last dose to surgery, median (IQR)	42.0 (33.0, 53.0)	43.0 (34.0, 55.0)	33.0 (29.0, 42.0)	**0.01**
Procedure				
Lobectomy/Bilobectomy	241 (87.3%)	215 (88.5%)	26 (78.8%)	0.06
Pneumonectomy	12 (4.3%)	8 (3.3%)	4 (12.1%)	
Segmentectomy/Wedge	23 (8.3%)	20 (8.2%)	3 (9.1%)	
Bronchoplasty				
Sleeve Resection	5 (1.8%)	5 (2.1%)	0 (0.0%)	0.57
Suture Closure	3 (1.1%)	3 (1.2%)	0 (0.0%)	
Angioplasty				
Suture/patch	3 (1.1%)	3 (1.2%)	0 (0.0%)	0.66
Stapled	3 (1.1%)	3 (1.2%)	0 (0.0%)	
Reoperation	9 (3.3%)	7 (2.9%)	2 (6.1%)	0.33
Institution				
Center #1	8 (2.9%)	4 (1.6%)	4 (12.1%)	**< 0.001**
Center #2	22 (8.0%)	8 (3.3%)	14 (42.4%)	
Center #3	15 (5.4%)	8 (3.3%)	7 (21.2%)	
Center #4	66 (23.9%)	66 (27.2%)	0 (0.0%)	
Center #5	75 (27.2%)	75 (30.9%)	0 (0.0%)	
Center #6	90 (32.6%)	82 (33.7%)	8 (24.2%)	

MIS – minimally invasive surgery; IQR- interquartile range; BMI- body mass index; FEV1- forced expiratory volume in one second; DLCO- diffusing capacity of carbon monoxide; PET- positron emission tomography; SUVmax- maximum standardized uptake value; EBUS- endobronchial ultrasound; VATS- Video-assisted Thoracic Surgery

**Table 2:** Pathologic results and treatment response by approach

Tumor characteristics	All (*N* = 276)	RATS (*N* = 243)	VATS (*N* = 33)	*p*-Value
Size [cm], median (IQR)	2.7 (1.5, 4.1)	2.7 (1.7, 4.0)	2.2 (0.0, 4.1)	0.22
Histology				
Adenocarcinoma	170 (61.6%)	143 (58.8%)	27 (81.8%)	**0.04**
Squamous Cell	92 (33.3%)	87 (35.8%)	5 (15.2%)	
Neuroendocrine/ Mixed/Other	14 (5.1%)	13 (5.3%)	1 (3.0%)	
Grade				
Well differentiated	9 (7.8%)	9 (8.6%)	0 (0.0%)	0.55
Moderately differentiated	52 (44.8%)	46 (43.8%)	6 (54.5%)	
Poorly/un-differentiated	55 (47.4%)	50 (47.6%)	5 (45.5%)	
**Lymph Node Harvest**				
LN count, median (IQR)	10.5 (3.0, 15.0)	11.0 (2.0, 15.0)	8.0 (5.0, 13.0)	0.88
N1	4.0 (2.0, 7.0)	4.0 (2.0, 7.0)	3.0 (1.0, 8.0)	0.24
N2	5.0 (2.0, 8.0)	5.0 (2.0, 8.0)	5.0 (3.0, 6.0)	0.94
Number of N2 Stations				
<3 Stations	71 (26.3%)	61 (25.7%)	10 (30.3%)	0.58
3 + Stations	199 (73.7%)	176 (74.3%)	23 (69.7%)	
**Pathologic Stage**				
Pathologic T-Stage				
ypT0	118 (42.8%)	108 (44.4%)	10 (30.3%)	0.30
ypT1	81 (29.3%)	70 (28.8%)	11 (33.3%)	
ypT2	44 (15.9%)	36 (14.8%)	8 (24.2%)	
ypT3	24 (8.7%)	20 (8.2%)	4 (12.1%)	
ypT4	9 (3.3%)	9 (3.7%)	0 (0.0%)	
Pathologic N-Stage				
ypN0	214 (77.5%)	189 (77.8%)	25 (75.8%)	0.93
ypN1	25 (9.1%)	21 (8.6%)	4 (12.1%)	
ypN2a	28 (10.1%)	25 (10.3%)	3 (9.1%)	
ypN2b	9 (3.3%)	8 (3.3%)	1 (3.0%)	
Resection Margin				
R0	264 (95.7%)	232 (95.5%)	32 (97.0%)	0.69
R1	12 (4.3%)	11 (4.5%)	1 (3.0%)	
**Treatment Response**				
Treatment effect present	241 (87.6%)	208 (86.0%)	33 (100.0%)	**0.02**
MPR	158 (57.9%)	142 (59.2%)	16 (48.5%)	0.24
pCR	118 (43.2%)	108 (45.0%)	10 (30.3%)	0.11
Lymph node downstaging				
cN1-2 > ypN0	112 (42.3%)	97 (41.8%)	15 (45.5%)	0.69

MIS – minimally invasive surgery; IQR- interquartile range; MPR – major pathologic response; pCR – comp

### Conversion to thoracotomy

**Fig. 1:**
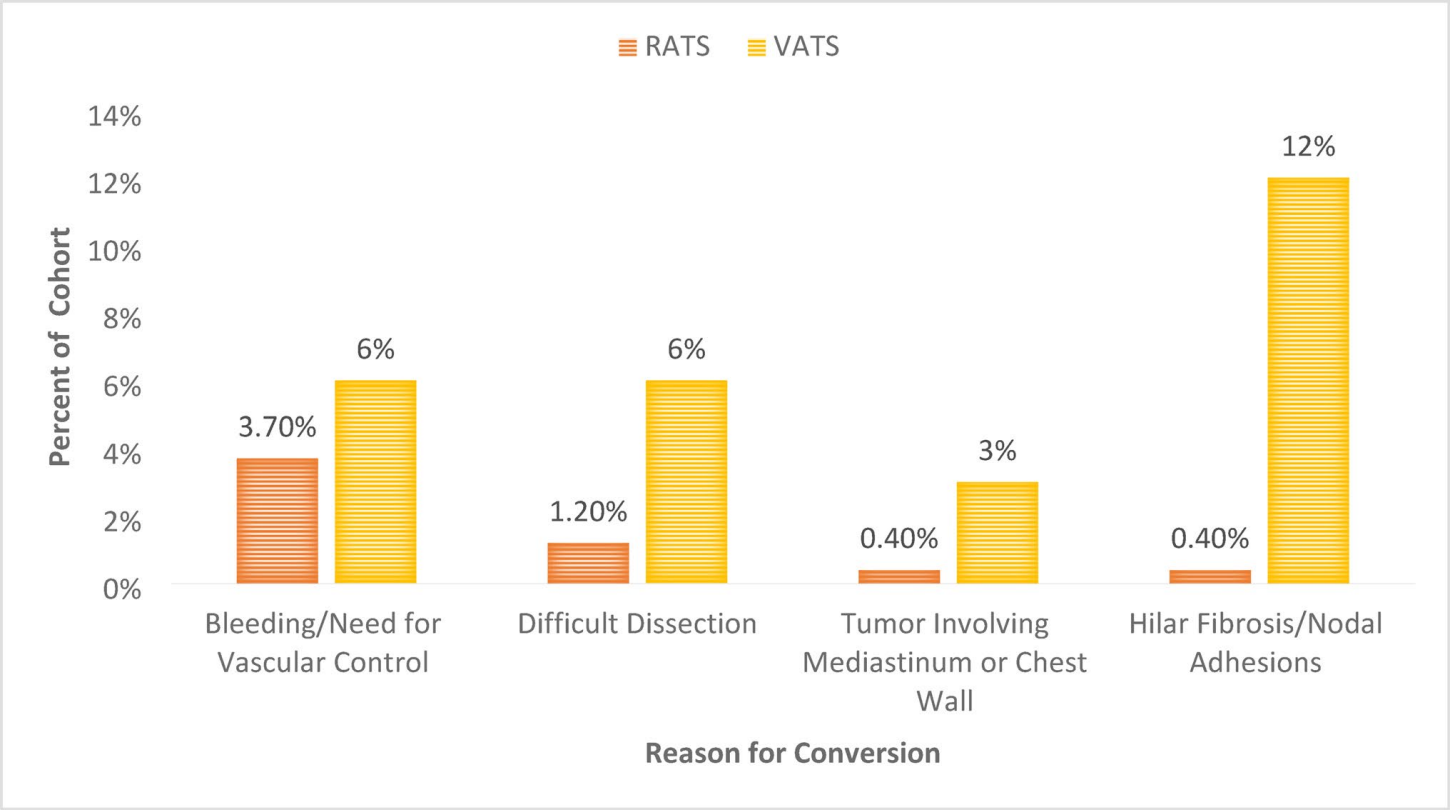
Reasons for conversion to thoracotomy by surgical approach

The conversion rate to thoracotomy was significantly higher in the VATS group (27.3% vs. 5.8%, *p* < 0.001). The most common reasons for conversion (Fig. 1) were vascular control or dense hilar fibrosis and challenging anatomy impeding dissection. Conversion for bleeding or the need for proximal vascular control was the most common reason for conversion in robotic cases. In contrast, conversions driven by difficult dissection were more often observed in VATS cases (Figure 1). Complex procedures including sleeve resection (*n* = 8) and vascular angioplasty (*n* = 6) were performed exclusively in the RATS group. Following IPTW adjustments, RATS remained associated with significantly fewer conversions to thoracotomy (*p* = 0.0045; Table 3).

**Table 3:** Unadjusted and adjusted post-operative outcomes by approach

	Without IPTW, No. (%)				With IPTW*, %		
	All (*N* = 276)	RATS (*N* = 243)	VATS (*N* = 33)	*p*-value	RATS (*N* = 243)	VATS (*N* = 33)	*p*-Value
**Operating Room Time** [min], median (IQR)	214.0 (152.5, 273.0)	216.0 (153.0, 279.0)	196.0 (146.0, 236.0)	0.09	231.2 (7.6)	205.4 (11.7)	0.07
**Estimated Blood Loss** [ml], median (IQR)	50.0 (25.0, 100.0)	50.0 (25.0, 100.0)	100.0 (50.0, 200.0)	**0.004**	92.2 (10.6)	156.3 (40.9)	0.13
**Conversion to Thoracotomy**	23 (8.3%)	14 (5.8%)	9 (27.3%)	**< 0.001**	7.2%	26.1%	**0.005**
**Length of Stay** [days], adjusted mean (SE)	4.8 (4.56)	4.7 (4.69)	5.3 (3.43)	**0.02**	4.6 (0.3)	5.0 (0.6)	0.46
**Postoperative Events**							
Any complication	98 (35.5%)	87 (35.8%)	11 (33.3%)	0.78	35.8%	34.5%	0.91
Major Complication	26 (9.4%)	19 (7.8%)	7 (21.2%)	**0.01**	7.4%	22.7%	**0.01**
**Pulmonary Complications**							
Prolonged air leak	44 (15.9%)	37 (15.2%)	7 (21.2%)	0.38	15.0%	23.4%	0.32
Atelectasis req. bronchoscopy	10 (3.6%)	8 (3.3%)	2 (6.1%)	0.42	3.5%	10.2%	0.16
Pleural effusion req. drainage	8 (2.9%)	7 (2.9%)	1 (3.0%)	0.96	2.0%	6.5%	0.26
Pneumonia	13 (4.7%)	11 (4.5%)	2 (6.1%)	0.70	4.2%	10.5%	0.21
Respiratory Failure	13 (4.7%)	9 (3.7%)	4 (12.1%)	**0.03**	3.9%	10.5%	0.10
**Cardiovascular Complications**							
Atrial fibrillation	17 (6.2%)	17 (7.0%)	0 (0.0%)	0.12	6.8%	0.0%	
Myocardial infarction	0 (0.0%)	0 (0.0%)	0 (0.0%)		0 (0.0%)	0 (0.0%)	
Pulmonary Embolus	1 (0.4%)	1 (0.4%)	0 (0.0%)	0.71	0 (0.0%)	0 (0.0%)	
Chylothorax	3 (1.1%)	3 (1.2%)	0 (0.0%)	0.52	1.3%	0.0%	
**Return to the Operating Room**	6 (2.2%)	2 (0.8%)	4 (12.1%)	**< 0.001**	1.0%	14.9%	**< 0.001**
Hemorrhage	4 (1.4%)	2 (0.8%)	2 (6.1%)	**0.02**	0.8%	5.8%	**0.03**
**Mortality within 60 days**	2 (0.7%)	2 (0.8%)	0 (0.0%)	0.60	0.8%	0.0%	

IPTW – Inverse probability of treatment weights; SE- standard error

* IPTW weights adjusted for age, body mass index, forced expiratory volume in one second, performance status, coronary artery disease, diabetes, clinical T-stage, and extent of resection

## Pathologic characteristics and treatment response

Median tumor size was similar between robotic and VATS groups (2.7 cm [IQR 1.7, 4.0] vs. 2.2 cm [IQR 0.0, 4.1]; *p* = 0.22). Adenocarcinoma was more frequent in the VATS group (81.8%) compared to RATS (58.8%) (*p =* 0.04). Tumor grade distribution and extent of lymph node harvest, including total lymph nodes and N1/N2 subsets, did not differ significantly between groups (Table 2). The majority of patients had pathologic T1 or T2 tumors, and ypN0 nodal status was noted in 77.5% of cases, with no significant differences by approach. Complete (R0) resection was accomplished in over 95% of patients in both groups (*p =* 0.93*)*. Evidence of treatment effect was observed in 87.6% of specimens, although a small subset (14.0%) of RATS cases had no detectable effect, compared to 0% in the VATS group (*p =* 0.02). Median percentage of residual viable tumor was low across the entire cohort (0.0%, IQR 0.0, 0.5%) and similar between groups (*p =* 0.22). Major pathologic response (MPR) occurred in 57.9% of patients and pathologic complete response (pCR) in 43.2%, without significant differences between approaches (*p =* 0.24 and *p =* 0.11, respectively). Lymph node downstaging from cN1–2 to ypN0 occurred in 42.3% of patrients, and was similar between RATS and VATS (*p =* 0.69).

## Postoperative outcomes

In the unadjusted analysis, median operating room time was slightly shorter for VATS (196 min [IQR 146, 236]) compared to RATS (216 min [IQR 153, 279], *p* = 0.09), while estimated blood loss was significantly lower in the RATS group (50 mL [IQR 25, 100] vs. 100 mL [IQR 50, 200], *p =* 0.004). Overall complication rates were similar between groups (35.8% RATS vs. 33.3% VATS, *p =* 0.78); however, major complications were more common with VATS (21.2% vs. 7.8% for RATS, *p =* 0.01). Respiratory failure (12.1% VATS vs. 3.7% RATS, *p =* 0.03) and return to the operating room (12.1% VATS vs. 0.8% RATS, *p <* 0.001) were significantly more frequent in the VATS cohort. Other individual complications, such as prolonged air leak, atelectasis requiring bronchoscopy, pneumonia, or pleural effusion, did not differ significantly. Sixty-day mortality was low across both approaches (0.8% RATS vs. 0% VATS, *p =* 0.60).

The IPTW adjusted mean length of stay was similar for RATS (4.6 days) than VATS (5.0 days, *p =* 0.46). The IPTW adjusted rates for major complications (7.4% VATS vs. 22.7% RATS, *p* = 0.01), and unplanned reoperation (1.0% vs. 14.9%, *p* < 0.001) were significantly lower for RATS compared to VATS. Differences in overall complication rates remained nonsignificant (35.8% vs. 34.5%, *p* = 0.91), as were rates of prolonged air leak, atelectasis, pleural effusion, pneumonia, atrial fibrillation, and 60-day mortality (Table 3). The previously observed advantage in respiratory failure and length of stay was attenuated and no longer statistically significant after adjustment (Table 3).

## Discussion

This multicenter study provides a contemporary comparison of real world outcomes between robotic-assisted and video-assisted thoracoscopic surgery after neoadjuvant chemoimmunotherapy for patients with non-small cell lung cancer. Our results demonstrate that RATS and VATS achieved a similarly high rate of complete resection and oncologic clearance, with no compromise in pathologic outcomes. However, RATS was associated with a markedly lower thoracotomy conversion rate and fewer severe complications. These findings underscore that a minimally invasive approach remains feasible even in complex post-induction cases and suggest potential advantages of the robotic platform in this setting.

The high success rate of RATS after neoadjuvant chemo-immunotherapy in centers with experience in minimally invasive surgery is an important finding. Only 5.8% of RATS cases required unplanned conversion to open thoracotomy, versus 27.3% of VATS cases (nearly a five-fold difference). This disparity aligns with emerging evidence from other series. For instance, Zeng et al. reported conversion rates of 7.5% for RATS and ~ 28% for VATS after neoadjuvant chemoimmunotherapy [10], closely mirroring our results. By contrast, earlier trials and experiences saw much higher rates of primary open thoracotomy following induction therapy [4, 8, 9, 15]. In the phase III CheckMate 816 trial, minimally invasive surgery was attempted in only about 30% of patients and conversion to open was required in ~ 11% of those receiving chemoimmunotherapy (versus 16% after chemotherapy alone) [4]. Small single-institution series with VATS have documented conversion rates up to 40–50% in locally advanced cases after immunotherapy, often attributable to dense hilar fibrosis and adhesions [13, 16]. Comparatively, the low overall conversion rate in this study provides a more desirable benchmark. The robotic platform, in particular, appears to facilitate completing difficult resections thoracoscopically. All complex sleeve lobectomies and several resections requiring pulmonary angioplasty in our cohort were successfully done with RATS, whereas none were attempted via VATS. This suggests that surgeons favored RATS for more challenging resections and were able to avoid thoracotomy even in anatomically demanding cases. Thus, the robotic approach may expand the boundaries of minimally invasive lung cancer surgery after induction therapy, enabling definitive resection without conversion in situations where traditional VATS might reach its limits.

Short-term postoperative outcomes in our study were also favorable for the robotic approach. Overall complication rates were similar between RATS and VATS, consistent with prior reports that adding immunotherapy does not significantly increase perioperative morbidity [9, 11, 13]. However, RATS was associated with a lower incidence of serious complications. Major complications occurred in only 7.8% of RATS patients, compared to 21.2% of VATS patients, translating to a nearly threefold reduction. In particular, life-threatening issues such as postoperative respiratory failure and bleeding requiring re-intervention were notably less frequent with RATS. The unplanned reoperation rate was 0.8% for RATS versus 12.1% for VATS. These differences suggest that the robotic technique may mitigate some surgical trauma and improve intraoperative control, thereby reducing the cascade of severe postoperative events. Moreover, patients in the RATS cohort experienced a modestly faster recovery, with a median hospital length of stay one day shorter than the VATS cohort. Faster recovery after RATS might stem from less frequent conversions to open surgery and lower major complication rates, as patients avoid the added pain and inflammation of thoracotomy and invasive reinterventions. Taken together, these results indicate that when performed by experienced teams, RATS offers at least equivalent if not improved postoperative outcomes compared to conventional VATS in the context of neoadjuvant therapy. Both approaches demonstrated low 60-day mortality in our series, underscoring the overall safety of lung resection following chemoimmunotherapy in specialized centers.

Operative efficiency and resource utilization were comparable between the two minimally invasive techniques, with some advantages observed for RATS. We found no significant difference in operative duration: median operation times were approximately 3.5 h for RATS and 3.3 h for VATS. This equivalence in surgical time is notable, as robotic cases included more complex resections involving bronchoplasty or angioplasty in 6% of cases. From a resource-use perspective, minimizing blood loss and avoiding unplanned conversions or reoperations can translate into lower overall utilization of healthcare resources (such as blood bank products, intensive care unit admissions, and prolonged hospital stays). While we did not perform a cost analysis, it is worth noting that the robotic system itself entails higher direct costs and specialized instruments. However, these costs might be partly offset if RATS leads to fewer complications, shorter hospitalization, and less need for escalation of care [17]. Our findings of equivalent operative times and potentially less postoperative morbidity with RATS suggest that, in an experienced setting, the robotic approach can achieve efficiency on par with VATS while conferring some intraoperative advantages.

This study has several limitations that must be considered when interpreting the results. First, the retrospective, non-randomized design is subject to selection biases. The choice of RATS or VATS was not randomized but rather determined by surgeon or center preference and resource availability. This imbalance reflects real-world practice patterns but also limits direct comparability. We attempted to mitigate confounding through inverse probability of treatment weighting, and the persistence of key findings (such as lower conversion and major complication rates with RATS) after adjustment strengthens our confidence in the results. Nonetheless, unmeasured differences in patient selection or tumor characteristics could still influence the outcomes. Another limitation is the relatively small size of the VATS subgroup, which reduces statistical power for certain comparisons and might mask subtle differences or risks unique to the thoracoscopic approach. Similarly, institutional clustering and platform adoption patterns represent important limitations of this study. Two centers performed exclusively robotic resections during the study period, reflecting institutional preference and resource allocation rather than absence of VATS capability. While all participating institutions are experienced thoracic surgery programs, unmeasured surgeon-specific factors and center-level effects cannot be fully accounted for in a retrospective design. Therefore, the observed differences between approaches should be interpreted cautiously and viewed as reflective of real-world practice patterns rather than definitive evidence of intrinsic platform superiority. Our follow-up was focused on immediate postoperative outcomes. Future studies are warranted to assess long-term oncologic results based on operative approach and surgical quality. Finally, our findings emanate from high-volume thoracic centers with significant expertise in minimally invasive techniques and thus may not be generalizable to all practice settings. The benefits of RATS noted here may depend on a certain level of surgical experience and access to advanced technology. Future studies will be valuable to validate these observations.

## Conclusions

In summary, our comparative analysis demonstrates that both RATS and VATS are viable approaches for NSCLC resection following neoadjuvant chemoimmunotherapy, but the robotic technique offers distinct advantages in this context. RATS was associated with a significantly lower risk of conversion to open surgery and fewer major postoperative complications, while achieving equivalent oncologic resection quality. These results support the feasibility of an expanded role for robotic lung resection after induction therapy, especially for complex cases that might otherwise necessitate thoracotomy. While further research is warranted to corroborate these findings and assess long-term outcomes, our data contribute to a growing body of evidence that the robotic minimally invasive approach can help surmount the technical challenges posed by neoadjuvant chemoimmunotherapy, ultimately enabling more patients to benefit from both modern systemic therapy and minimally invasive surgery.

## Electronic Supplementary Material

Below is the link to the electronic supplementary material.

Supplementary Material 1

## Data Availability

Data is provided within the manuscript.

## Abbreviations

AUC: Area under the curve
BMI: Body mass index
EBUS: Endobronchial ultrasound
FDG: Fluorodeoxyglucose
NSCLC: Non-small cell lung cancer
PET: Positron emission tomography
pCR: Pathologic complete response
MIS: Minimally invasive surgery
MPR: Major pathologic response
ICI: Immune checkpoint inhibitor
IQR: Interquartile range
IPTW: Inverse probability of treatment weighing
VATS: Video-assisted thoracoscopic surgery
