# Supplementary material for: Multicenter study comparing outcomes of robotic versus video-assisted thoracoscopic resection of non-small cell lung cancer following neoadjuvant chemoimmunotherapy
Source: J Robot Surg. 2026 Apr 2;20(1):398. doi: 10.1007/s11701-026-03326-4 (PMC13046649; doi:10.1007/s11701-026-03326-4)
Supplement: Supplementary file 1 — Supplementary Material 1 [file 11701_2026_3326_MOESM1_ESM.docx]

**Supplemental Table 1:** Sensitivity analyses excluding centers that exclusively performed RATS

|  | **With IPTW*, %** | | |
| --- | --- | --- | --- |
|  | **RATS** | **VATS** | ***p*-Value** |
|  | **(N = 102)** | **(N = 33)** |  |
| **Operating Room Time** | 163.7 (8.2) | 203.6 (13.6) | **0.013** |
| [min], median (IQR) |  |  |  |
| **Estimated Blood Loss** | 141.7 (43.6) | 157.8 (34.1) | 0.77 |
| [ml], median (IQR) |  |  |  |
| **Conversion to Thoracotomy** | 15.07% | 24.33% | 0.28 |
| **Length of Stay** [days], | 5.4 (0.5) | 5.2 (0.6) | 0.84 |
| adjusted mean (SE) |  |  |  |
| **Postoperative Events** |  |  |  |
| Any complication | 36.08% | 36.60% | 0.96 |
| Major Complication | 8.06% | 25.21% | **0.02** |
| **Pulmonary Complications** |  |  |  |
| Prolonged air leak | 15.28% | 24.80% | 0.28 |
| Atelectasis req. bronchoscopy | 4.67% | 8.51% | 0.49 |
| Pleural effusion req. drainage | 2.08% | 6.12% | 0.34 |
| Pneumonia | 5.01% | 11.17% | 0.30 |
| Respiratory Failure | 4.03% | 12.11% | 0.14 |
| **Cardiovascular Complications** |  |  |  |
| Atrial fibrillation | 2.35% | 0.00% |  |
| Myocardial infarction | 0 (0.0%) | 0 (0.0%) |  |
| Pulmonary Embolus | 0 (0.0%) | 0 (0.0%) |  |
| Chylothorax | 0.90% | 0.00% |  |
| **Return to the Operating Room** | 0.00% | 17.42% | **< 0.01** |
| Hemorrhage | 1.13% | 6.99% | 0.09 |
| **Mortality within 60 days** | 0.00% | 0.00% |  |

**Supplemental Table 2:** Study Site Information

|  | **Center #1** | **Center #2** | **Center #3** | **Center #4** | **Center #5** | **Center #6** |
| --- | --- | --- | --- | --- | --- | --- |
| Number of cases contributed | 8 | 22 | 15 | 66 | 75 | 90 |
| Region | Germany | US | US | US | US | France |
| Average number of anatomic lung resection per year  (2022–2024) | 190 | 249 | 135 | 186 | 264 | 332 |
| MIS approach used | VATS and Robotic | VATS and Robotic | VATS and Robotic | Robotic | Robotic | VATS and Robotic |
| Subset of MIS for stage I lung cancer | 99% | 98.1% | 98% | 99% | 99% | > 95% |
| Date of first patient resected after neoadjuvant chemo-immunotherapy  included | 09/2023 | 07/2021 | 7/2019 | 08/2021 | 9/2022 | 9/2020 |

MIS – minimally invasive surgery; VATS- Video-assisted Thoracic Surgery
